# Supplementary material for: DMRforPairs: identifying Differentially Methylated Regions between unique samples using array based methylation profiles
Source: BMC Bioinformatics. 2014 May 15;15:141. doi: 10.1186/1471-2105-15-141 (PMC4046028; doi:10.1186/1471-2105-15-141)
Supplement: Additional file 2 — DMRforPairs output for the comparison of A431-MCF7 and NA17018-NA17105. Please start from the HTML files in each folder. Available via the BMC Bioinformatics website. [file 1471-2105-15-141-S2.zip › 1394847754114233_MOESM2_ESM/A431_MCF7/figures/25474.pdf]

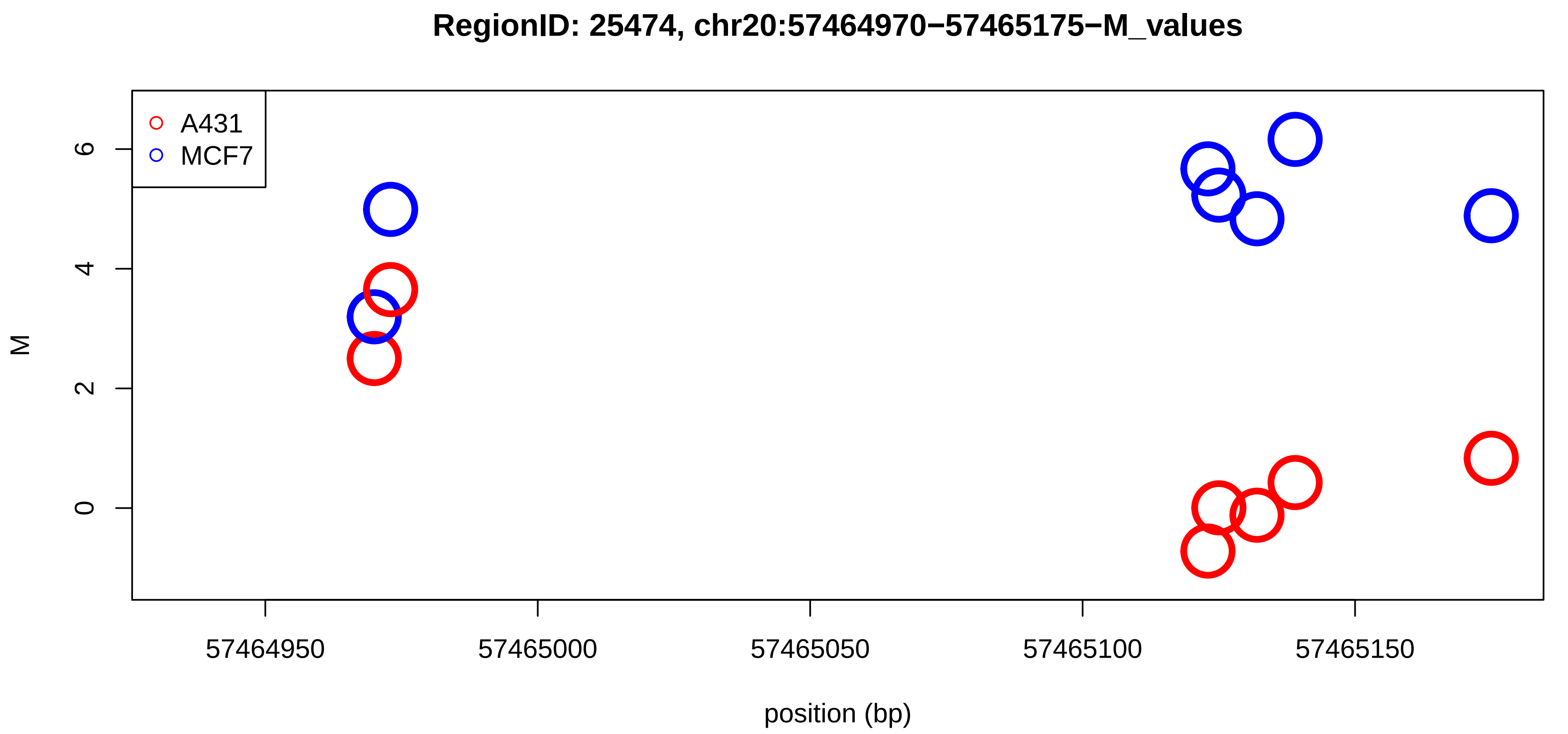
RegionID: 25474, chr20:57464970−57465175−M_values
A431
MCF7
M
0
2
4
6
57464950
57465000
57465050
57465100
57465150
position (bp)

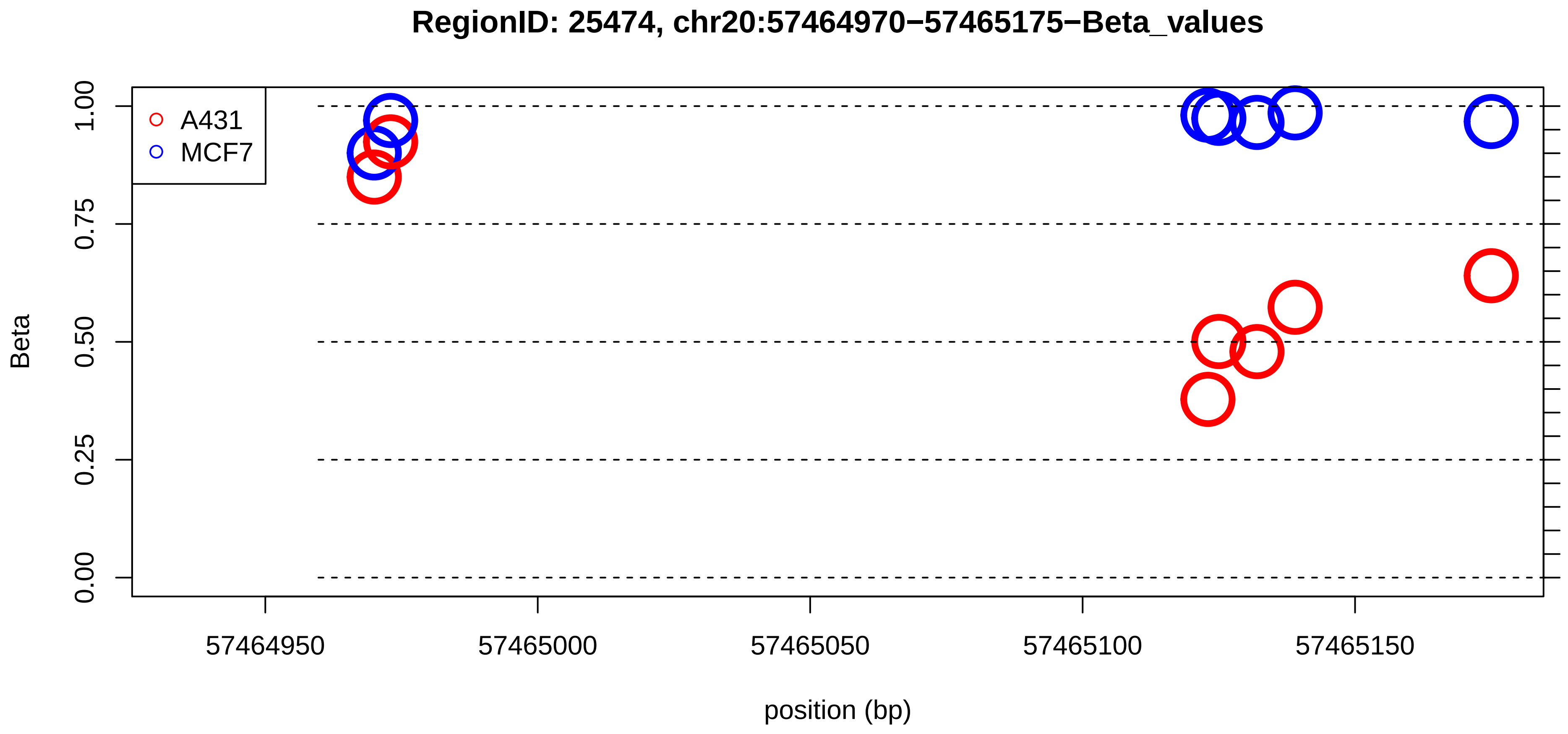
RegionID: 25474, chr20:57464970−57465175−Beta_values
A431
MCF7
Beta
0.00
0.25
0.50
0.75
1.00
57464950
57465000
57465050
57465100
57465150
position (bp)

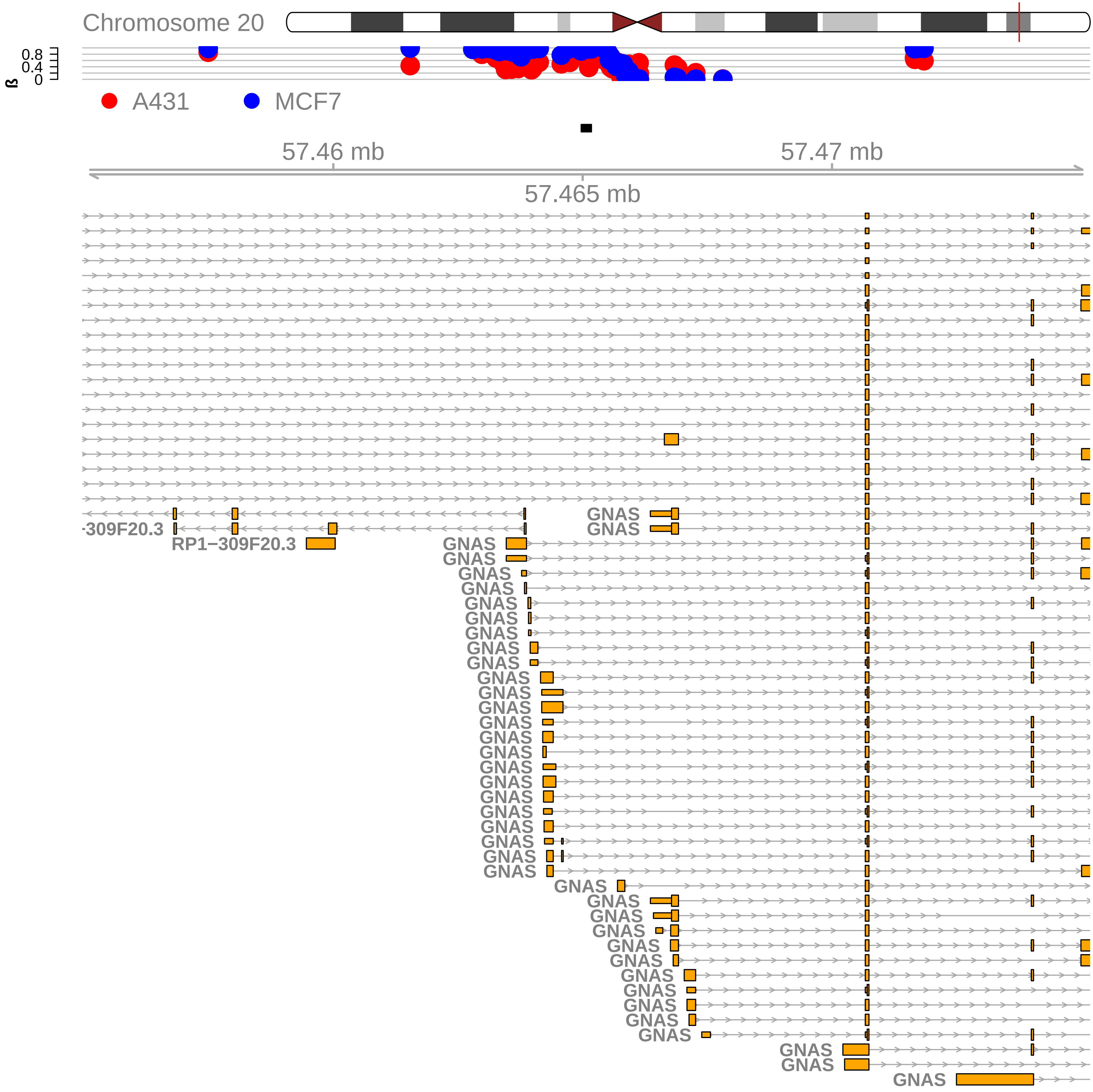
Chromosome 20
0.8
0.4
0
β
A431
MCF7
57.46 mb
57.47 mb
57.465 mb
-309F20.3
RP1−309F20.3
GNAS
